# Rapid and Selective NH_3_ Sensing by Porous CuBr

**DOI:** 10.1002/advs.201903390

**Published:** 2020-02-16

**Authors:** Andreas T. Güntner, Markus Wied, Nicolay J. Pineau, Sotiris E. Pratsinis

**Affiliations:** ^1^ Particle Technology Laboratory Department of Mechanical and Process Engineering ETH Zurich Sonneggstrasse 3 Zurich 8092 Switzerland

**Keywords:** breath analysis, environmental monitoring, gas sensors, semiconductors, wearables

## Abstract

Fast and selective detection of NH_3_ at parts‐per‐billion (ppb) concentrations with inexpensive and low‐power sensors represents a long‐standing challenge. Here, a room temperature, solid‐state sensor is presented consisting of nanostructured porous (78%) CuBr films. These are prepared by flame‐aerosol deposition of CuO onto sensor substrates followed by dry reduction and bromination. Each step is monitored in situ through the film resistance affording excellent process control. Such porous CuBr films feature an order of magnitude higher NH_3_ sensitivity and five times faster response times than conventional denser CuBr films. That way, rapid (within 2.2 min) sensing of even the lowest (e.g., 5 ppb) NH_3_ concentrations at 90% relative humidity is attained with outstanding selectivity (30–260) over typical confounders including ethanol, acetone, H_2_, CH_4_, isoprene, acetic acid, formaldehyde, methanol, and CO, superior to state‐of‐the‐art sensors. This sensor is ideal for hand‐held and battery‐driven devices or integration into wearable electronics as it does not require heating. From a broader perspective, the process opens exciting new avenues to also explore other bromides and classes of semiconductors (e.g., sulfides, nitrides, carbides) currently not accessible by flame‐aerosol technology.

## Graphical abstract

Porous and nanostructured CuBr films are prepared by flame‐aerosol deposition of CuO and resistance‐monitored dry‐conversion to Cu and eventually CuBr. Excellent NH_3_ sensitivity down to 5 ppb at high relative humidity and outstanding selectivity over ethanol, acetone, H_2_, CH_4_, isoprene, acetic acid, formaldehyde, methanol, and CO is obtained at room temperature, ideal for low‐power devices in environmental monitoring or breath analysis.

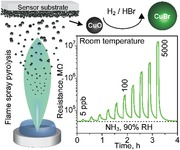

## 1 Introduction

Ammonia (NH_3_), a major industrial commodity (142 Mt in 2017^1^), is toxic as well as a tracer for food spoilage detection^2^ and putative breath marker for impaired kidney^3^ and liver function (e.g., cirrhosis,^4^ hepatic encephalopathy,^4^ or injury^5^). As a result, there is a strong interest in developing reliable sensors over a wide range of NH_3_ concentrations: from 250 to 2900 ppb^6^ in mouth‐exhaled breath down to few ppb in indoor air^7^ at high relative humidity (RH). Also, NH_3_ is present in gas mixtures containing a myriad of compounds^8^ requiring also high selectivity.

Several materials have been proposed for room‐temperature NH_3_ sensing including organic diodes,^5^ polymers like polyaniline^9^ or PEDOT:PSS nanowires^10^ and carbon‐based graphene oxide,^11^ 3D sulfonated reduced graphene oxide hydrogels^12^ or polyaniline‐carbon nanotube composites.^13^ All these show slow response and recovery times,^9^ limited selectivity^10^, ^12^, ^13^ or sensitivity^11^, ^12^ to detect relevant parts‐per‐billion (ppb) concentrations while realistic humidities have been rarely considered.

Most promising as a solid‐state and low‐cost sensor material is CuBr featuring high selectivity and sensitivity to low NH_3_ concentrations (e.g., 20 ppb^14^) also at room temperature.^15^ The CuBr is an ionic conductor with Cu^+^ as charge carrier. Upon exposure to NH_3_, these Cu^+^ ions are immobilized by forming stable ammine complexes, e.g., $\text{Cu}{\left({\text{NH}}_{3}\right)}_{2}^{+}$.^16^ Thin CuBr films have been fabricated by radio frequency sputtering of CuBr,^16^ liquid‐phase bromination of Cu films in solutions^17^ and direct evaporation and condensation of CuBr where also CeO_2_ overlayers were prepared to mitigate humidity interference.^14^ However, these methods usually result in micron‐sized CuBr particles^14^ and rather dense film morphologies^15^, ^17^ that could impede efficient NH_3_ diffusion in the film and interaction with the CuBr surface.

**Figure 1:**
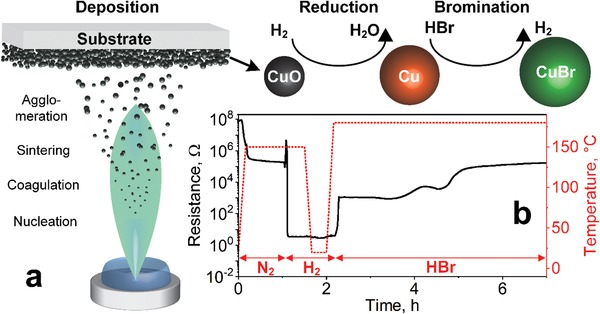
a) Schematic of the three‐step CuBr gas sensing film assembly by flame‐aerosol deposition and dry conversion: First, CuO nanoparticles are made by flame spray pyrolysis and deposited by thermophoresis as highly porous film onto cooled Al_2_O_3_ substrates with interdigitated Pt electrodes. Subsequently, the CuO nanoparticles are reduced by H_2_ and brominated by HBr gas to form the CuBr films. b) In situ monitored resistance of the film (solid line, left ordinate) upon reduction in H_2_ (*t* = 1–2 h) and bromination in HBr (*t* = 2–7 h). The corresponding temperature profile is shown as dashed line (right ordinate).

Here, we introduce a novel fabrication route yielding highly porous and nanostructured CuBr films for improved NH_3_ sensitivity and fast response dynamics at room temperature and high RH. Such films are obtained by flame‐aerosol deposition^18^ of CuO nanoparticles onto interdigitated electrodes (**Figure** **1**a). Then, these films are reduced and brominated by a dry process monitored in situ^19^ through their resistance (Figure 1b). They are tested as room temperature sensors for NH_3_ down to 5 ppb at realistic 90% RH. Furthermore, their sensitivity is compared to that of denser CuBr films with comparable thickness made by conventional wet bromination.^17^ Finally, their NH_3_ selectivity over typical confounders in indoor air, breath, and meat spoilage monitoring (i.e., isoprene, ethanol, methane, acetone, hydrogen, acetic acid, methanol, formaldehyde, and CO) is evaluated.

## 2 Results and Discussion

### 2.1 Flame‐Aerosol Deposition of Porous CuO Films

**Figure 2:**
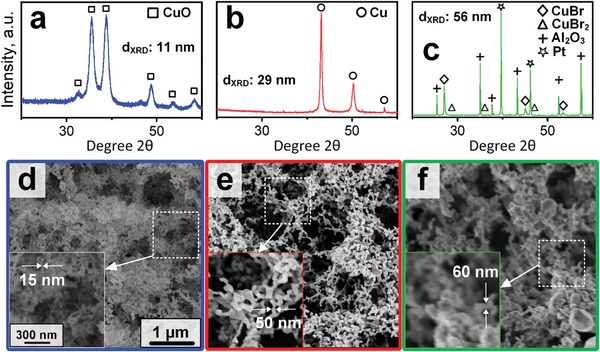
XRD patterns of powders (a and b) and when deposited as film (c) by flame spray pyrolysis as‐prepared (a), after the dry reduction (b) and bromination (c) together with top‐view SEM images of the corresponding films (d–f). Note that (a) and (b) were obtained from the powders as the XRD signals of the actual films were too weak. Reference peak positions for monoclinic CuO (squares), cubic Cu (circles), cubic CuBr (diamonds), monoclinic CuBr_2_ (triangles), cubic Pt (stars), and rhombohedral Al_2_O_3_ (crosses) are indicated together with crystal sizes of CuO (a), Cu (b), and CuBr (c), as calculated by Rietveld refinement from the XRD patterns. Note that the Pt and Al_2_O_3_ in c are associated to the substrate.

The CuO nanoparticles are prepared by flame spray pyrolysis (FSP, Figure 1a) of a liquid organometallic precursor.^20^ These particles are formed by nucleation and oxidation, before they coagulate and sinter to larger aggregates and agglomerates. They consist of cubic CuO with high crystallinity and average crystal size of 11 nm, as determined by X‐ray diffraction (XRD) of filter‐collected powder (**Figure** **2**a). Their average particle size measured by nitrogen adsorption is 10 nm and quite similar to the crystal size suggesting monocrystalline particles, consistent with literature.^20^

These fractal‐like CuO agglomerates are directly deposited^18^ onto sensor substrates (Figure 1a) resulting in a fine interconnected network with a wide range of pore diameters from few up to 500 nm, as seen with top‐view scanning electron microscopy (SEM, Figure 2d). With a higher magnification (inset), also the constituent (primary) nanoparticles become visible with dimensions similar to those obtained by N_2_ adsorption. X‐ray signal attenuation indicates an average film porosity of 94% ± 1% that is comparable to similarly prepared flame aerosol‐made SnO_2_ films.^18^ Such an open film morphology is attractive for gas sensing, as the analytes can easily penetrate into the film and interact with its large available surface.

### 2.2 Formation of CuBr Films

In order to preserve this sensor‐favorable film architecture, the CuO is converted by a dry process at moderate temperatures to mitigate sintering and structure collapse: first by reduction to Cu with H_2_ at 150 °C for 30 min and subsequently by bromination to CuBr with HBr at 180 °C for 5 h (Figure 1a). The overall conversion is monitored in situ by resistance readout^19^ exploiting the distinctly different bulk resistivities^21^ of Cu, CuO, and CuBr,^22^ i.e., ≈10^−6^, 10^4^, and 10^5^ Ω m at 20 °C, respectively. Figure 1b shows the film resistance (solid line) and temperature (dashed line) during this dry conversion. At room temperature, the resistance of the CuO film is ≈90 MΩ indicating an interconnected network between the substrate‐mounted electrodes,^19^ in agreement with SEM (Figure 2d). This drops to 0.2 MΩ when heating the film to 150 °C for 1 h in N_2_ (dashed line) due to thermal activation of intrinsic charge carriers^23^ in the semiconductive CuO.

When exposed to H_2_, the resistance rapidly drops to just 4 Ω and remains quite stable for 1 h suggesting reduction to a conductive Cu network. In fact, XRD of similarly reduced CuO powders indicates solely cubic Cu crystals (Figure 2b, circles). Upon reduction, the Cu crystals and particles grew to 29 and 58 nm, respectively, suggesting polycrystallinity and/or extended particle necking. This growth is observed also in the corresponding films (Figure 2d,e). Most importantly, however, the characteristic lace‐like structure and open morphology of flame‐aerosol deposited films^18^ is preserved during conversion with similar porosity for the resulting Cu film (i.e., 95% ± 1%). However, more macropores (i.e., >50 nm) are now visible (Figure 2e).

Upon the onset of dry bromination, the film resistance jumps to 1 kΩ and stays rather constant for 1 h (Figure 1b). Thereafter, it increases further up to 0.4 MΩ during the following 5 h (*t* = 2 – 7 h), indicating the formation of a semiconductive network. In general, the film resistances of CuBr are lower than that of CuO, despite its higher bulk resistance as described above. This may be due to the higher temperature of the CuBr film (180 vs 150 °C, dashed line Figure 1b) but also smaller grain boundary resistances due to larger particle size and extended inter‐particle necking (Figure 2f vs 2d) should contribute. Remarkably, the Cu bromination takes significantly longer than the CuO reduction. This may be associated to the lower HBr concentration (ca. 0.1 mol%) during bromination compared to that of H_2_ (100 mol%) during reduction. But also the larger initial particle diameter of the Cu particles (i.e., 58 nm) than the CuO (i.e., 10 nm) for the reduction may play a role, resulting in a longer solid‐state diffusion pathway. Furthermore, the larger ionic size of bromine versus oxygen should result in lower diffusivity in the respective particles.

The gradual increase of the film resistance during bromination suggests a diffusion‐limited transformation following a “shrinking core” model.^24^ First, a CuBr layer is formed rapidly at the particle's surface, resulting in the observed sudden resistance increase (to 1 kΩ), while the core remains metallic Cu. Thereafter, the core is slowly converted to CuBr through solid‐state diffusion, as suggested by the almost continuous increase of the film resistance. The origin of the slight resistance decrease after 2 h (i.e., *t* = 4 h) of bromination could be attributed to formation of unwanted hygroscopic and water‐soluble CuBr_2_. The formation of CuBr_2_ was observed during sputtering by XRD that compromised the stability of such films.^16^ However, XRD (Figure 2c) of the CuBr films on the Al_2_O_3_ substrates indicates the presence of only cubic CuBr (diamonds) while no CuBr_2_ (triangles) is detected. During bromination, the average crystal size grew to 56 nm. This should be related to the crystal volume expansion during bromination while sintering of neighboring particles is also possible.

### 2.3 CuBr Film Morphology and Comparison to Other Fabrication Methods

The corresponding SEM top‐view image of a CuBr film is shown in Figure 2f with a higher magnification of a selected area (dashed frame) as inset. Most importantly, the rather porous and open morphology of the CuO and Cu films (Figure 2d,e) is preserved also during dry bromination. However, the porosity is decreased to 78% ± 3% and also the structural dimensions are larger than those of the Cu film, in line with XRD and BET (Figure 2b versus 2c). In specific, the diameters of single particles range from about 30 to 500 nm. Note that Figure 2f is slightly blurry, probably due to electric charging of the films during SEM.

**Figure 3:**
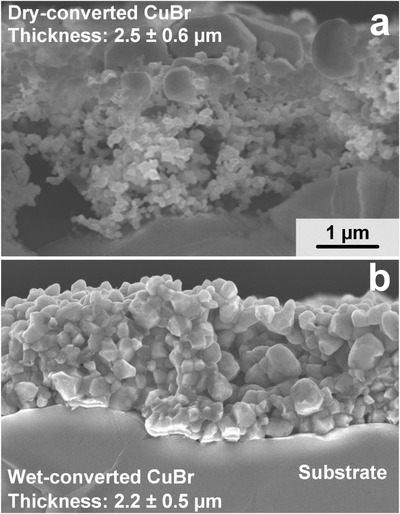
Cross‐sectional SEM of the dry‐ (a) and wet‐converted (b) CuBr films. Average thicknesses and STD are indicated.

**Figure** **3**a shows a cross section of such a dry‐converted CuBr film of 2.5 ± 0.6 (standard deviation (STD)) µm with a vertically inhomogeneous morphology. More specifically, the top layers are denser with some large (up to 1 µm) CuBr particles, as observed already by top‐view SEM (Figure 2f). The lower layers consisting of much finer structures of CuBr nanoparticles are more porous with even extended voids (e.g., bottom‐left of Figure 3a). This is particularly interesting as the lower layers usually dominate gas sensing being in the vicinity of the electrodes. Such a nanostructured and highly porous film morphology is unprecedented for CuBr films. Other fabrication methods result usually in significantly larger average particle sizes, e.g., 650–3380 nm for films made by thermal deposition through CuBr evaporation and condensation.^14^ Similar or even larger particles were obtained by wet bromination of Cu in solutions or radio frequency sputtering of CuBr featuring also more compact film morphologies.^17^

To investigate such morphological effects on gas sensing more closely, we fabricated also CuBr films by wet bromination. Therefore, the same flame aerosol‐deposited and reduced Cu films (Figure 2e) were immersed into a CuBr_2_ solution (instead of the dry conversion with HBr above, please see Experimental Section for details). These films consisted of pure CuBr (Figure S1, diamonds, Supporting Information) with average crystal size of 134 nm. A cross‐sectional SEM image of the resulting film is shown in Figure 3b having an average thickness of 2.2 ± 0.5 µm, comparable to the dry‐converted one (Figure 3a). Most importantly, however, its morphology is significantly denser than the dry‐converted one consisting of coagulated and larger CuBr nanoparticles, in line with literature.^17^ The wet‐converted film porosity is 43% ± 6% that is significantly lower than the dry‐converted film (i.e., 78% ± 3%).

### 2.4 NH_3_ Sensing at Room Temperature

**Figure 4:**
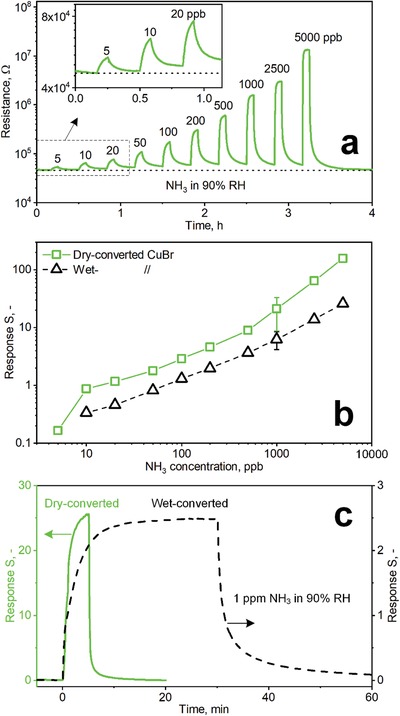
a) Sensor film resistance of dry‐converted CuBr films upon exposure to 5–5000 ppb NH_3_ at 90% RH. b) Corresponding responses of the dry‐ (squares) and wet‐converted (triangles) films. Note that 5 ppb NH_3_ is not detectable with some wet‐converted films. Symbols and error bars at 1 ppm NH_3_ indicate the average response and STD of three identically produced sensors. c) Response over time of a dry‐ (solid line, left ordinate) and wet‐converted (dashed line, right ordinate) film. Note the different ordinate scales.

The dry‐converted CuBr films were tested for sensing of 5–5000 ppb NH_3_ at 90% RH (**Figure** **4**a). When exposed to 5000 ppb of NH_3_, the resistance rapidly increases from 47 kΩ to 13 MΩ corresponding to a response (S) of 276. When exposed to NH_3_, the Cu^+^ as charge carriers are immobilized by forming^16^ $\text{Cu}{\left({\text{NH}}_{3}\right)}_{2}^{+}$ that results in the observed resistance increase. Remarkably, this interaction is rapid and reversible even at room temperature, as indicated by the full recovery of the initial resistance baseline (dashed line, Figure 4a) and in line with literature.^14^

Most impressively, NH_3_ concentrations down to 5 ppb (see also inset of Figure 4a for higher magnification) are detected and can be distinguished clearly from 10 and 20 ppb. This highlights the excellent sensing properties of these flame aerosol‐deposited and dry‐converted CuBr films. The signal‐to‐noise‐ratio (SNR, >70) is remarkable and should enable the detection of even lower concentrations with an extrapolated lower limit of detection (LOD) of 210 parts‐per‐trillion (ppt) considering a typical SNR of 3. This is superior to state‐of‐the‐art room temperature NH_3_ sensors: The lowest detected NH_3_ concentration is 10 ppb with organic diodes operated, however, under dry conditions.^5^ Polyaniline sensors detect 40 ppb NH_3_ at ≥90% RH,^9^ but these are usually for single‐use only as they recover too slowly. Other polymers like PEDOT:PSS nanowires feature higher LODs of 100 ppb,^10^ which is even higher for carbon‐based reduced graphene oxide (1.2 ppm in dry air)^11^ and 3D sulfonated reduced graphene oxide hydrogels (1.5 ppm).^12^ Finally, metal‐oxides can also detect sub‐ppm NH_3_ concentrations (e.g., Si‐doped MoO_3_ with LODs of 51 ppb at 90% RH^25^ and pure MoO_3_ of 280 ppt in dry air^26^) but these require typically elevated operational temperatures (e.g., 400–450 °C).

### 2.5 Morphology Effects on NH_3_ Sensing

Figure 4b shows the NH_3_ sensor responses of the wet‐ (triangles) and dry‐converted (squares) CuBr for NH_3_ between 5 to 5000 ppb at 90% RH. The latter features significantly higher responses than the former for all NH_3_ concentrations due to their larger specific surface area,^27^ finer particle sizes and inter‐particle structures (Figure 3a vs 3b). In fact, chemoresistive semiconductors show dramatically increased responsiveness when approaching structural dimensions of the space charge width (roughly twice the Debye length).^28^ For polycrystalline CuBr, the Debye length is in the order of 10 nm (at 400 K).^29^ As a result, the preservation of nanoscaled (i.e., <100 nm) dimensions in the film layers near the electrodes might be an advantage of the dry‐converted CuBr films. Even though flame‐made gas sensors exhibit remarkable stability,^25^ the long‐term performance of these dry‐converted CuBr films needs to be evaluated. No degradation of the sensing layer was observed, at least, for the wet‐converted CuBr films during 80 h of continuous operation (Figure S2, Supporting Information).

Figure 4c shows the responses over time for the dry‐ (solid, left ordinate) and wet‐converted (dashed, right ordinate) CuBr films to 1 ppm NH_3_ at 90% RH. The response and recovery times of the dry‐converted films are 2.2 min and 50 s while those of the wet‐converted ones are 10 and 6 min, respectively. Note that both responses approach steady state. For higher magnification of the dry‐converted film with marked response and recovery times, please see Figure S3, Supporting Information. In principle, the faster response and recovery times of these dry‐converted films could be attributed to their higher porosity (78% vs 43%) and smaller crystal size (Figure 2c vs Figure S1, Supporting Information) than the wet‐converted films. The higher porosity can facilitate faster diffusion of NH_3_ through the sensing film while the smaller crystal size can lead to shorter solid‐state diffusion length when forming $\text{Cu}{\left({\text{NH}}_{3}\right)}_{2}^{+}$ upon interaction of NH_3_ with CuBr.^16^ To accurately determine the reason for the better performance of dry‐converted sensors, however, a systematic investigation^19^ of the impact of film characteristics on sensor performance through its process synthesis variables is needed.

### 2.6 Selectivity

**Figure 5:**
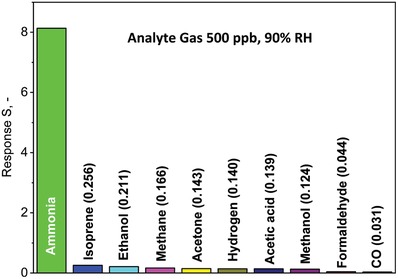
Response of dry‐converted CuBr films to 500 ppb of breath‐ and indoor air‐relevant gases at 90% RH.

The dry‐converted CuBr sensor was tested also with isoprene, ethanol, methane, acetone, hydrogen, acetic acid, methanol, formaldehyde, and carbon monoxide (CO), all at a concentration of 500 ppb and 90% RH (**Figure** **5**). Most remarkably, the sensor responds strongest to NH_3_ with selectivity > 30 with CO being the highest one (>260). This is similar to thermally deposited CuBr films with a CeO_2_ overlayer^14^ for some of these analytes.

Other NH_3_ sensors feature lower selectivities to these analytes. In fact, polymer‐based PEDOT:PSS nanowires^10^ are quite sensitive to ethanol (NH_3_ selectivity ≈15 vs CuBr 38 from Figure 5) while composites of polyaniline and multi‐walled carbon nanotubes^13^ showed only moderate selectivity over acetone (7 vs CuBr 57 from Figure 5). Also metal‐oxides like pure or Si‐MoO_3_ possess only moderate selectivity over acetone (3.6).^25^ While this can be improved by combining distinctly selective sensors in rather orthogonal^30^ sensor arrays that possess excellent NH_3_ accuracy (21 ppb) when monitoring human breath and skin emissions,^31^ the complexity and cost increase. As single sensor, only organic diodes seem to have competitive selectivities to ethanol and acetone, though this needs to be confirmed under high humidity.^5^

In some cases of breath analysis or indoor air quality monitoring, even the NH_3_ selectivity of CuBr may be insufficient. In fact, the selectivity to hydrogen and acetone is ≈60, but both can reach exhaled breath concentrations of tens of ppm (e.g., acetone during ketogenic diet^32^ or H_2_ in case of carbohydrate intolerance^33^). Also ethanol (selectivity ≈40) may be present at high ppm concentrations in the background of hospital air from disinfectants or cleaning agents.^34^ However, this can be improved by combining the CuBr sensor with microporous membranes^35^ or sorption columns (e.g., packed beds of activated ceramics^36^ or polymers^37^) that can remove critical confounders.

## 3 Conclusion

Nanostructured porous and crystalline CuBr films were prepared by flame‐aerosol deposition of CuO onto interdigitated electrodes and subsequent dry reduction‐bromination. Tracking the film resistance enabled precise monitoring of the CuO conversion to CuBr while no undesired CuBr_2_ was formed. These films possessed excellent NH_3_ sensing properties to detect even 5 ppb (SNR > 70) at 90% RH. In comparison to compact CuBr films made by conventional wet‐conversion, the responses of the dry‐converted ones were an order of magnitude stronger while their response and recovery times were more than five times faster. Both were attributed to the nanostructured porous morphology of dry‐converted CuBr films facilitating rapid gas transport through them and enhanced resistance modulation. Most importantly, these films exhibited outstanding selectivity (30–260) over various breath‐ and indoor‐relevant confounders (isoprene, ethanol, methane, acetone, hydrogen, acetic acid, methanol, formaldehyde, and CO) that seems superior to most state‐of‐the‐art NH_3_ sensors.

From a broader perspective, combining flame‐aerosol deposition with dry conversion creates exciting opportunities to access other bromides and even new classes of chemoresistive semiconductors (e.g., sulfides, nitrides, carbides). This addresses a major bottleneck of current flame‐made gas sensors that are restricted to metal oxides. With this process, unique sensing material compositions can be found that exhibit selectivity to key molecules already at room temperature. At the same time, the key advantages of flame‐aerosol‐deposition are maintained, most importantly, the scalable synthesis of highly porous films for high sensitivity and fast response times. This is a drawback of denser sensing films made by conventional methods (e.g., screen‐printing). In addition, all process steps can be performed in situ on the chip level to minimize the number of operational units, which is attractive for industry. This can contribute to the synthesis of new low‐power gas sensors that are urgently sought in portable breath analyzers^38^ and detectors for distributed air^39^ and food quality monitoring networks.^40^

## 4 Experimental Section

##### CuO Preparation

Copper oxide nanoparticles were produced by FSP.^41^ In brief, the liquid precursor consisted of Deca Copper 8 (Borchers, Germany) dissolved in a 2:1 volumetric mixture of 2‐ethylhexanoic acid (Sigma–Aldrich, 99%, Switzerland) and xylene (Sigma–Aldrich, 99%, Switzerland) at a total metal ion concentration of 0.25 m.^20^ The solution was supplied to the FSP burner at 4 mL min^−1^ and dispersed with 5 L min^−1^ O_2_ at a pressure drop of 1.6 bar. This fine spray was ignited with a premixed and ring‐shaped CH_4_/O_2_ flame (1.25/3.25 L min^−1^) and sheathed by an O_2_ ring at 5 L min^−1^ to ensure full combustion. The particles were collected on a glass‐fiber filter (GF6 Albet‐Hanemuehle, 257 mm diameters) at 50 cm above the FSP burner by the help of a vacuum pump or directly deposited by thermophoresis^18^ for 9 min onto Al_2_O_3_ sensor substrates (electrode type #103, Electronic Design Center, Case Western Reserve University, USA) mounted on a water‐cooled substrate holder 20 cm above the nozzle. These substrates featured interdigitated Pt electrodes and a Pt resistance temperature detector (RTD) on the front and a Pt heater on the back. Lastly, the mechanical stability of the nanoparticulate film was improved by in situ annealing^42^ at 14.5 cm above the nozzle with a particle‐free xylene flame at 11 mL min^–1^ and dispersed with 5 L min^−1^ oxygen.

##### CuBr Conversion

The CuO films on the substrates were converted to CuBr by gas‐phase reduction and bromination. Therefore, the CuO loaded sensor substrates were mounted on Macor holders and installed in a Teflon chamber.^43^ The sensor substrates were heated by applying a DC voltage (R&S HMC8043, Germany) to the substrate heater and controlled by the RTD connected to a multimeter (Keithley, Integra Series 2700, USA). Additionally the film resistance between the interdigitated electrodes was measured in situ^19^ (Keithley, Integra Series 2700, USA). First, the sensors were heated to 150 °C in inert N_2_ (20 mL min^−1^, Pangas 5.0) and kept for 1 h before switching to pure H_2_ (20 mL min^−1^, Pangas 4.5) for 30 min. Thereafter, films were cooled down to room temperature for 30 min in H_2_. For bromination, the temperature was increased to 180 °C and the gas was switched to a HBr mixture (20 mL min^−1^, Pangas, 1040 ppm HBr in N_2_ 5.0) for 5 h. All gases were provided from calibrated gas cylinders with high‐resolution mass flow controllers (MFCs, Bronkhorst, Netherlands) using inert Teflon tubing. For further analysis, also filter‐collected CuO nanoparticles from the FSP process were identically reduced to the sensor films in an Autochem reactor (Micromeritics) with a gas flow of 40 mL min^−1^.

For comparison, CuBr films were prepared with a conventional wet‐phase bromination in a solution.^17^ Therefore, Cu films were fabricated as described above and placed in 5 mL of a 0.1 M CuBr_2_ (Sigma–Aldrich, 99.999%, Switzerland) in methanol (Sigma–Aldrich, ≥99.9%, Switzerland) solution for 1 min. Afterward the sensor substrate was rinsed with pure methanol (Sigma–Aldrich, ≥99.9%, Switzerland) for 30 s.

##### Powder and Film Characterization

Crystal phases were determined by XRD obtained with a Bruker AXS D8 Advance XRD (Bruker, USA) operated at 40 kV and 30 mA at 2θ = 20 − 80° with a scanning step size of 1.45 × 10^−2^° and a scanning time per step of 1 s. Materials and lattice parameter alterations were identified by peak allocation and peak shift identification with reference structural parameters of monoclinic CuO (PDF 78‐0428), cubic Cu (04‐0836), cubic CuBr (77‐1997), monoclinic CuBr_2_ (45‐1063), cubic Pt (87‐0642), and rhombohedral Al_2_O_3_ (75‐1865) using the software Diffrac.eva V3 (Bruker, USA). Crystal sizes are determined through the Rietveld fundamental parameter method using the software TOPAS (Bruker, USA). The specific surface areas (SSA) of the powders were measured by nitrogen adsorption (Micromeritics II Plus, USA) using the Brunauer–Emmett–Teller (BET) method. The particle sizes were calculated assuming spherical particles with the corresponding material densities (CuO 6.31 g cm^−3^, Cu 8.96 g cm^−3^, CuBr 4.71 g cm^−3^).

The film morphology was analyzed by SEM with a Hitachi S‐4800 FE‐SEM operated at 5 kV for top‐view and 7 kV for cross‐sectional images. Film thicknesses were obtained with the software ImageJ from >100 measurements for each film. Film porosities were calculated by X‐ray signal attenuation of the substrates' Al_2_O_3_ peaks due to the nanoparticulate film.^18^ Therefore, the peak intensity at 25.6, 35.1, 43.3, 52.5, and 57.5° were measured of the bare (*I_in_*) and covered (*I_em_*) substrate. The following exponential attenuation law was applied:^44^

(1) $$\frac{I_{em}}{I_{in}}=\;exp\left[-\frac{\mu }{\rho_{s}}\rho_{s}\;s_{s}\right]$$

with µ/ρ_*s*_ being the mass attenuation coefficient obtained from the XCOM Photon Cross Section Database,^45^ *ρ_s_* the aforementioned material density, and *s_s_* the average film thickness determined by SEM.

##### Gas Testing

Sensing performance of the CuBr films was evaluated at room temperature by using identical holders and chambers as for reduction/bromination. Gas mixtures were prepared with a mixing setup described in detail elsewhere.^25^ In brief, analyte gases (i.e., NH_3_, isoprene, ethanol, methane, acetone, hydrogen, acetic acid, methanol, formaldehyde, and CO) were supplied from calibrated cylinders (10 or 50 ppm in synthetic air, PanGas) and dosed to dry synthetic air (Pangas 5.0, C_n_H_m_ and NO_x_ ≤ 100 ppb) with calibrated mass flow controllers. Humidity was added by bubbling dry synthetic air through distilled water and admixing it to the analyte flow. The total flow was 1 L min^−1^. All transfer lines and the sensor chamber were made of inert Teflon to minimize analyte gas adsorption and heated to 55 °C to avoid water condensation, as done in breath samplers (e.g., for mass spectrometry^46^ and chemoresistive sensors^43^). Note that droplet formation can lead to short circuiting and dissolution of the CuBr sensing film by NH_4_OH formation. Due to the heating, the temperature of the sensor substrate was 40.9 ± 0.2°C (Figure S4a, green line, Supporting Information), as measured with the substrate's resistance temperature detector (RTD) and sufficiently high above typical indoor and human body temperatures. The gas temperature just before the sensor chamber was 37.9 ± 0.1 °C (Figure S4a, black line, Supporting Information) that reduced the RH from 90.9% ± 0.3% to 31.1% ± 0.1% (Figure S4b, Supporting Information), as measured with a SHT2x sensor (Sensirion AG, Switzerland). The sensor film resistance was continuously monitored with a multimeter (Keithley, Integra Series 2700, USA). Sensor response was defined as:

(2) $$S=\frac{R_{analyte}}{R_{air}}-1$$

with *R_analyte_* and *R_air_* being the film resistances with analyte and in air, respectively. Response and recovery times were defined as the time to reach or recover 90% of the resistance change.

## Conflict of Interest

The authors declare no conflict of interest.

## Supporting information

Supporting Information
