# Supplementary material for: Rapid and Selective NH3 Sensing by Porous CuBr
Source: Adv Sci (Weinh). 2020 Feb 16;7(7):1903390. doi: 10.1002/advs.201903390 (PMC7140997; doi:10.1002/advs.201903390)
Supplement: Supplementary file 1 — Supporting Information [file ADVS-7-1903390-s001.pdf]

Copyright WILEY-VCH Verlag GmbH & Co. KGaA, 69469 Weinheim, Germany, 2020.

ADVANCED SCIENCE
Open Access

Supporting Information

for *Adv. Sci.,* DOI: 10.1002/advs.201903390

Rapid and Selective NH_3 Sensing by Porous CuBr

*Andreas T. Güntner, Markus Wied, Nicolay J. Pineau, and Sotiris E. Pratsinis**

Copyright WILEY-VCH Verlag GmbH & Co. KGaA, 69469 Weinheim, Germany, 2018.

Supporting Information

Rapid and selective NH_3 sensing by porous CuBr

*Andreas T. Güntner, Markus Wied, Nicolay J. Pineau and Sotiris E. Pratsinis**

Dr. A. T. Güntner, M. Wied, N. J. Pineau, Prof. S. E. Pratsinis
Particle Technology Laboratory, Department of Mechanical and Process Engineering, ETH Zurich, Sonneggstrasse 3, Zurich, 8092, Switzerland
E-mail: sotiris.pratsinis@ptl.mavt.ethz.ch

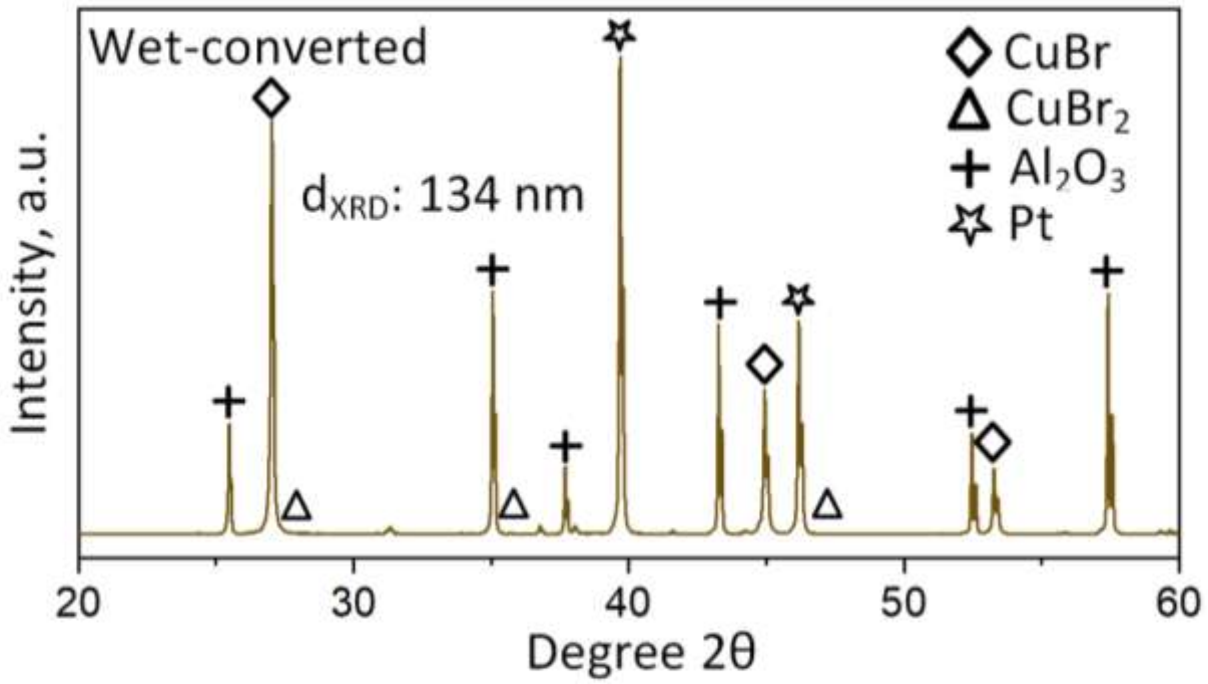

Figure S1. XRD pattern of the flame-deposited film, in situ annealed and converted by wet bromination. Reference peaks for cubic CuBr (diamonds), monoclinic $CuBr_2$ (triangles), cubic Pt (stars) and rhombohedral Al_2O_3 (crosses) are indicated together with the average crystal size of CuBr. Peaks from Pt and Al_2O_3 are from the sensor substrate.

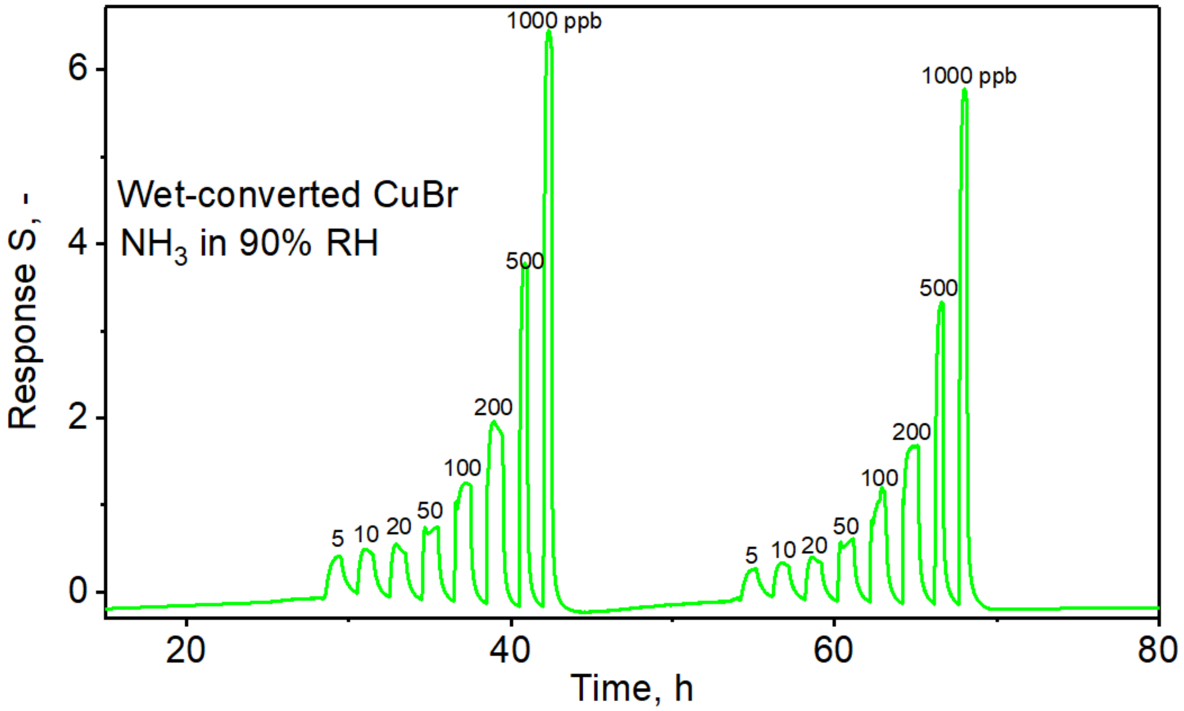

Figure S2. Sensor response of a wet-converted CuBr film when exposed twice to 5 - 1000 ppb NH_3 at 90% RH during 80 h of continuous operation.

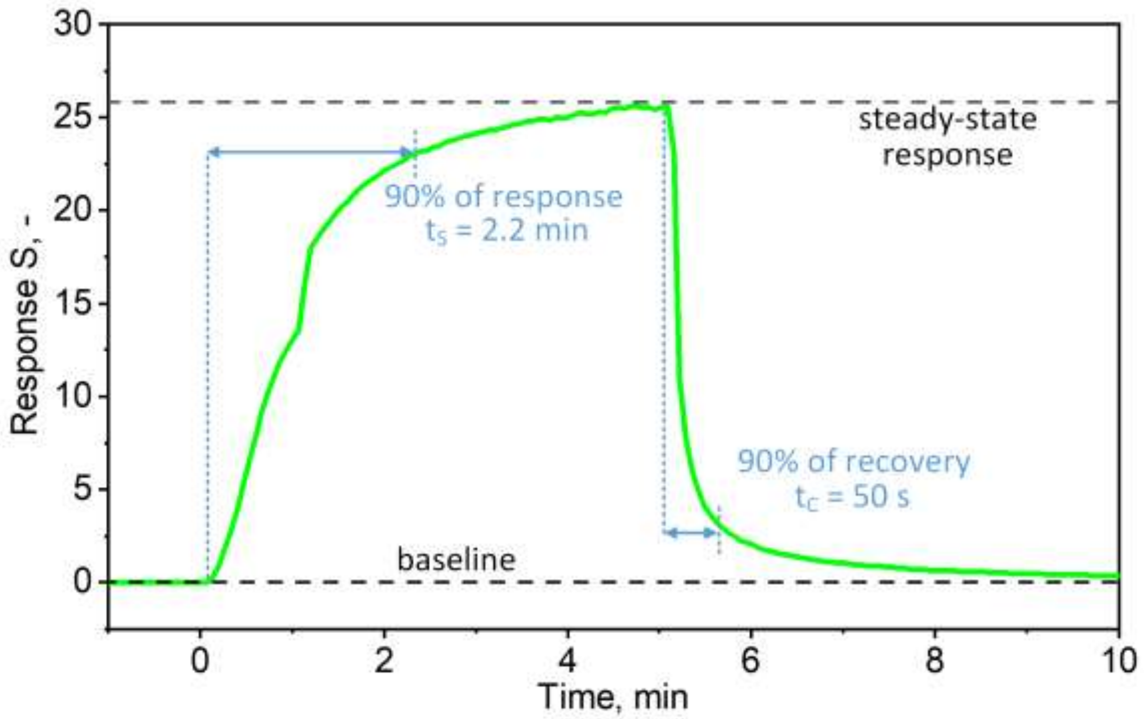

Figure S3. Sensor response over time of a dry-converted CuBr film. The baseline and steady-state response (dashed lines) are indicated together with the response and recovery times.

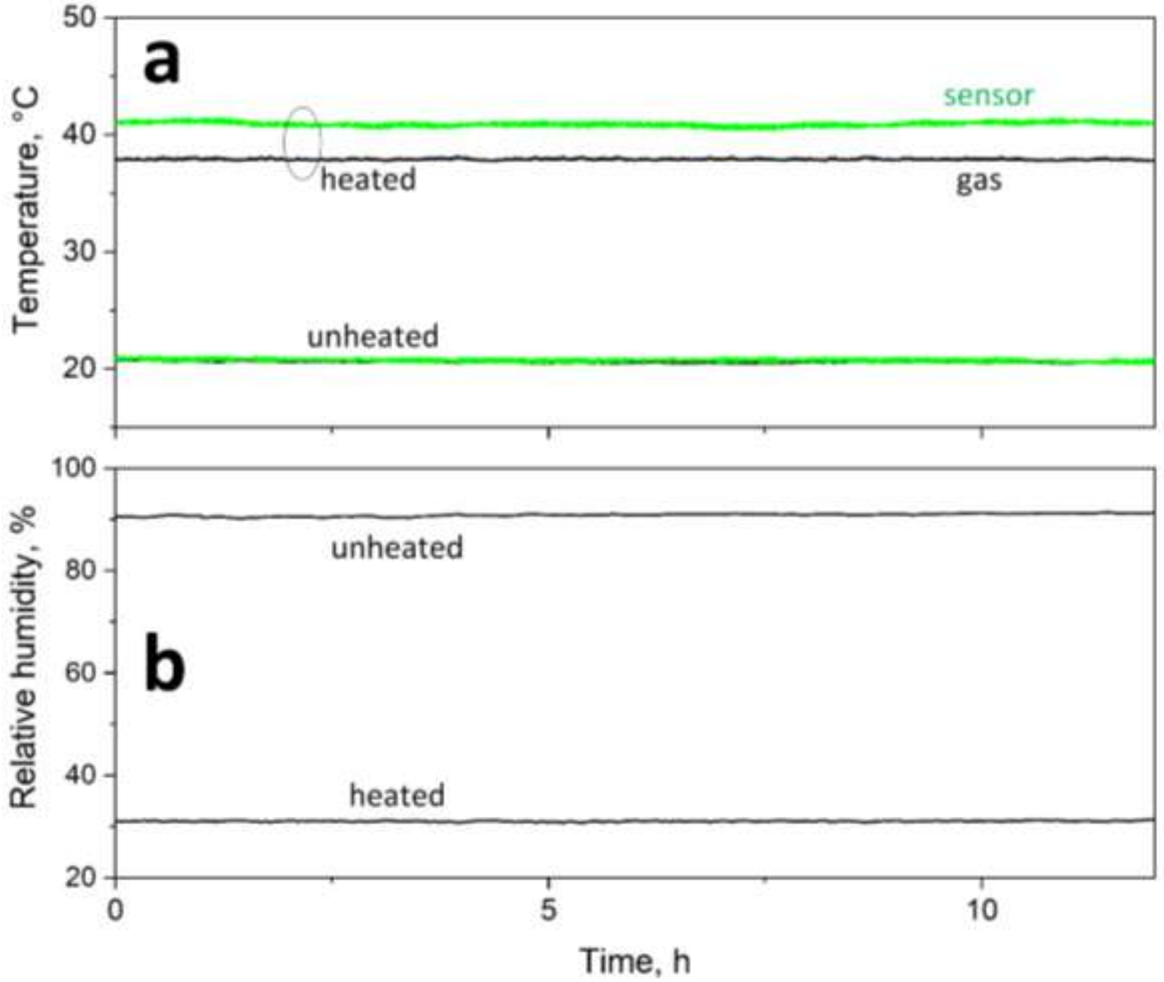

Figure S4. (a) Temperature of the sensor substrate (green lines) and gas (black lines) with and without heated chamber and transfer line to 55 °C at 1 L min^{-1} synthetic air flow and 90% RH during 12 h. Note that the temperature lines for gas and sensor substrate without heating are on top of each other, as expected. (b) Corresponding RH of the gas stream with heating.
